# On consciousness, resting state fMRI, and neurodynamics

**DOI:** 10.1186/1753-4631-4-S1-S9

**Published:** 2010-06-03

**Authors:** Arvid Lundervold

**Affiliations:** 1 Department of Biomedicine, Neuroinformatics and Image Analysis Laboratory, University of Bergen Jonas Lies vei 91, N-5009 Bergen, Norway; 2 Department of Radiology, Haukeland University Hospital, N-5021 Bergen, Norway

## Abstract

**Background:**

During the last years, functional magnetic resonance imaging (fMRI) of the brain has been introduced as a new tool to measure consciousness, both in a clinical setting and in a basic neurocognitive research. Moreover, advanced mathematical methods and theories have arrived the field of fMRI (e.g. computational neuroimaging), and functional and structural brain connectivity can now be assessed non-invasively.

**Results:**

The present work deals with a pluralistic approach to "consciousness'', where we connect theory and tools from three quite different disciplines: (1) philosophy of mind (emergentism and global workspace theory), (2) functional neuroimaging acquisitions, and (3) theory of deterministic and statistical neurodynamics – in particular the Wilson-Cowan model and stochastic resonance.

**Conclusions:**

Based on recent experimental and theoretical work, we believe that the study of large-scale neuronal processes (activity fluctuations, state transitions) that goes on in the living human brain while examined with functional MRI during "resting state", can deepen our understanding of graded consciousness in a clinical setting, and clarify the concept of "consiousness" in neurocognitive and neurophilosophy research.

## Background

During the last decade functional magnetic resonance imaging (fMRI) has been introduced as an experimental tool in the study of human consciousness, e.g. [1-8]. From the early report by Binder and coworkers on "conceptual processing" and "task-unrelated thoughts" captured by resting state fMRI [9], and the "default mode network" hypothesis by Raichle et al. [10], substantial improvements in MR image acquisition technology, experimental designs, and image analysis methodology have taken place. Functional MRI investigations now provide an increasingly important source of information to the modeling of integrative brain functions [11-16] and modern philosophy of mind, including the emergence of consciousness (e.g. [17,18]), and sophisticated mathematical and statistical models for fMRI signal processing and interpretation have come into play [19-30].

The present work deals with a pluralistic approach to "consciousness", where we try to connect theory and tools from three quite different disciplines:

1. *philosophy of mind (emergentism and global workspace theory),*

2. functional* neuroimaging* recordings, and

3. theory of deterministic and statistical* neurodynamics*

This in order to explore and review – surely prematurely and haltingly – an experimental and theoretical framework for the study of large-scale neuronal processes (activity fluctuations, state transitions) that goes on in the conscious human brain while examined with functional MRI during "rest".

The occurrence of state transitions in the brain (i.e. neurodynamics [31,32]) and the presence of noise in neuronal systems (e.g. [33]) have been jointly investigated and incorporated into the framework of *stochastic dynamical systems theory.* Stochastic dynamical systems theory (e.g. [34-36]) deals with the study of dynamical systems (discrete or continuous rule-based time evolutions on a state space) under the influence of noise. It has been shown that the coupling of noise to nonlinear deterministic equations of motion can lead to non-trivial effects such as stabilizing unstable equilibriums, transitions between coexisting deterministic stable states (attractors), and enhanced response of a nonlinear system to external signals (i.e. stochastic resonance). In our setting, perceptions, attention, and memories have extensively been modeled as state space attractors in dynamical neural networks (e.g. [37,38]). We want to explore these concepts and theoretical results in the context of resting state fMRI [39]. These are 4-D recordings consisting of spatial multivariate discrete time series (Δ*t* ~ 1-3 s) of length *T* (~ 4-6 min), expressing local magnetic BOLD (Blood-Oxygen-Level-Dependent) signal changes in a collection of *n* brain regions, and can be regarded as realizations of our neurodynamical system with state space ℝ*^n^*. In the simplest case, each state space component *x_i_* : {1,..., *T*} → ℝ, (*i* = 1,..., *n*) can be represented by a single brain voxel, consisting of thousands of neurons, or be represented by a larger spatial region as obtained from e.g. independent component analysis (spatial ICA). The joint activation pattern during the observation period *T*, where neuronal activities are embedded in noise, is denoted [*x*_1_(*t*),..., *x_n_*(*t*)]*_t_*_=1,…,_*_T _*, from which "neural correlates of consciousness" (NCC) [40-42] and dynamical state space models can be derived. This approach has a modest relationship to the recent introduction of ensemble dynamics and neural mass models into the imaging domain [43,44], concepts that have been pertinent to computational electrophysiology for many years [21,45,46].

The rest of the paper is organized as follows. In the next section we briefly introduce the concepts of 'emergentism' and the 'global workspace theory' which we have found particularly relevant regarding consciousness and the philosophy of mind in our empirical context. Next we present the exploding field of functional magnetic resonance imaging and so-called resting state functional connectivity MRI (rs-fcMRI) mapping, including the default mode network (DMN) as a particular subset of the resting state networks (RSNs) that can be computed from the 4-D fMRI recordings. We also refer to recent literature that apply these technologies in the study of 'consciousness' within basic cognitive neuroscience and in a clinical setting (vegetative state, sleep, anesthesia, etc.). The most widely used class of methods for analyzing resting state fMRI recordings (i.e. ICA) is presented in a separate section. This brings us into the theory of dynamical systems and neurodynamics and its application to time course data in voxels or in spatially more extensive ICA components representing functional networks with long-range dependencies. In an experimental study we present an illustrative example, where the fMRI data are taken from one individual in a collection of more than hundred subjects that participates in a comprehensive and longitudinal study of cognitive aging that includes structural and functional brain imaging, neuropsychological testing and genetic profiling. Finally, we give some concluding remarks and future perspectives.

To guide a more detailed and mature tour, we have deliberately provided references to relevant and recent literature in our cross-disciplinary endeavor.

## Theory and methods

### Philosophy of mind, emergentism, and global workspace theory

**Figure 1:**
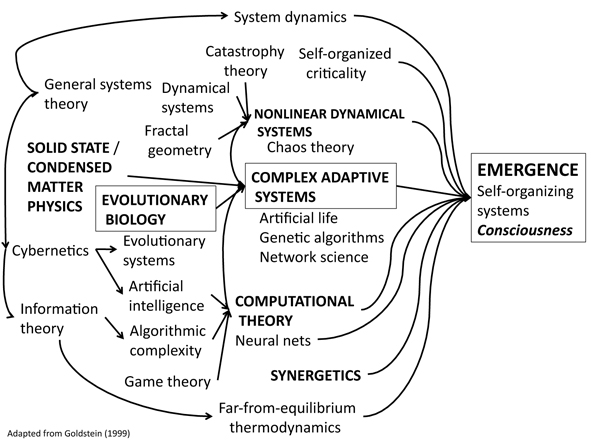
**Mathematical and scientific roots of emergence**, where the 'route to consciousness' is indicated by boxes (modified from [47]).

The concept of **'emergence'** can be defined as (J. Goldstein, 1999):

*... the way complex systems and patterns arise out of a multiplicity of relatively simpler interactions - occurring on the macro level, in contrast to the micro-level components and processes out of which they arise.* Hence, the construct of **'emergence'** (cf. Fig. 1 ) is applicable to the brain and the integrative levels of brain function in man and animal, including 'consciousness'.

According to [47], the common characteristics of 'emergence' are [here focussing on fMRI-derived **"emergents"** of resting state networks (RSNs) including the default mode network (DMN)]:

**Radical novelty**: emergents have features that are not previously observed in the complex system being studied.

**Coherence or correlation**: emergents appear as integrated wholes that tend to maintain some sense of identity over time. This coherence spans and correlates the separate lower-level components into a higher-level unity.

**Global or macro level**: the locus of emergent phenomena occurs at a global or macro level, in contrast to the micro-level locus of their components.

**Dynamical**: emergent phenomena are not pre-given wholes but arise as a complex system evolves over time. As a dynamical construct, emergence is associated with the appearance of new attractors in dynamical systems (i.e. bifurcation).

**Ostensive**: emergents are recognized by "showing themselves" [*in casu*: can be measured by fMRI].

In this neurophilosophical landscape, where resting state fMRI is an empirical ingredient, a particular relevant position is the viewpoint of **global workspace (GW) theory** or the "conscious access hypothesis" proposed by Bernard Baars [48]. According to this theory there is a global distribution of conscious content where multiple brain networks cooperate and compete. For such, Baars states the following theoretical claims [49]:

“1. *Conscious perception enables access to widespread brain sources; unconscious sensory processing is much more limited.*

2. *Conscious perception, inner speech, and visual imagery enable working memory functions; there is no evidence for unconscious access to working memory.*

3. *Conscious events enable almost all kinds of learning: episodic and explicit learning, but also implicit and skill learning.*

4. *Conscious perceptual feedback enables voluntary control over motor functions, and perhaps over any neuronal population and even single neurons.*

5. *Conscious contents can evoke selective attention.*

6. *Consciousness enables access to ‘self’ – executive interpreters, located in part in the frontal cortex.* ”

Since its inception in 1983, the global workspace theory has been refined and elaborated, integrating experimental data and models from cognitive psychology, artificial intelligence, electrophysiology, and neuroimaging [41,50-54]. Global workspace theory has thus evolved into a comprehensive framework for empirically based characterization and understanding of 'consciousness', and might also show to be a useful interpretative tool regarding neural correlates of consciousness using computational neuroimaging with resting state fMRI recordings (cf. [55] with commentaries, and [56,57]).

### Neuroimaging and resting state fMRI

... If we hope to understand how the brain operates, we must take into account the component that consumes most of the brain's energy: spontaneous neuronal activity.

From Fox & Raichle [39]

The observation that spontaneous BOLD fMRI activity is not random noise, but is specifically organized in the resting human brain as functionally relevant resting-state networks (RSNs) has generated a new avenue in neuroimaging and cognitive research.

**Figure 2:**
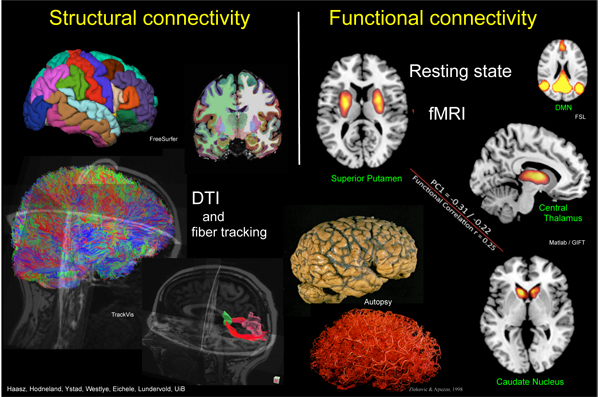
**Structural and functional connectivity** assessed with multimodal MRI (anatomical T1-weighted 3D scan, diffusion tensor imaging, BOLD fMRI).

Biswal et al. [58] were the first to demonstrate the feasibility of using fMRI to detect such spatially distributed networks within primary motor cortex during resting-state. From a MRI time course of 512 echo-planar images obtained every 250 ms in the resting human brain they calculated temporal correlations across the brain with the time-course from a seed voxel whose spatial location was chosen from a prior finger-tapping study. Time courses of low frequency (< 0.1 Hz) fluctuations where found to have a high degree of temporal correlation within sensorimotor regions and also with time courses in several other regions that can be associated with motor function, and they concluded that correlation of low frequency fluctuations in the resting state BOLD signal is a manifestation of functional connectivity of the brain. With increasing evidence during the last decade, the common understanding is that RSNs reflect interactions in cognitively relevant functional networks of the brain and are not, as early debated, a simple consequence of ongoing non-neuronal physiological processes such as the cardiac and respiratory cycles [59] (but see also [60,61]). Resting state functional connectivity MRI (rs-fcMRI), combined with structural connectivity mapping (cf. Fig. 2), has therefore proven to be a useful probe for functional alterations in the brain as a consequence of changes in brain state, disease processes, neurodevelopment and aging, pharmacological interventions, and genetics [62-71].

Moreover, several fMRI studies have demonstrated that some of these self-organizing, resting state networks coincide with brain regions that are found to be* deactivated* across several fMRI (and PET) studies where an external stimulus or a cognitive paradigm is applied. Thus, these brain locations are more active at rest than during task performance. These observations have led to the hypothesis that the brain remains active in an organized fashion during the resting state, denoted the* default mode network* (DMN) [10,39]. The default mode hypothesis has been extensively studied [10,71-78], including direct electrophysiological measurement of default network areas [79], and its changes in different clinical states of consciousness [8]. The concepts and exploration of RSNs and the DMN has therefore been regarded as* "a paradigm shift in functional brain imaging''*[80,81].

#### Spatial Independent Component Analysis (ICA) in the study of resting state fMRI

Independent component analysis (ICA) [82,83] has proven to be a powerful exploratory tool for data-driven analysis of fMRI recordings. It is used to blindly estimate distributed spatial patterns in the brain, jointly with hidden source processes in the observed data, under the assumptions of statistically independent patterns and non-Gaussianity of the source components [84,85]. In this setting, one typically represents the 4-D fMRI dataset as a *p* × *n* matrix **X**, where the *n* columns consist of an enumeration of the *n* voxels that covers the imaged 3-D object of interest (i.e. the brain), and the rows consist of the recorded signals in these voxels at *p* different time points. Furthermore, one assumes that a matrix decomposition exists such that **X = AS**, where **S** is a *p* × *n* matrix of* source signals,* and **A** is a *p* × *pmixing matrix*. This expresses that the ICA model is a* generative model* that describes how the observed data are generated by a mixing process of the components (i.e. row vectors) in **S**, and that the independent components are* latent variables* that cannot be directly observed. Both **A** and **S** must be estimated from **X** under some general statistical assumptions (independence and non-Gaussianity), and the ICA method is thus a type of* blind source separation* (BSS).

During ICA estimation, the matrix **S** is optimized to contain, in its rows, statistically independent areas in the brain (spatial maps, or components), each with an internally consistent time course (the corresponding column vector in **A**).The observed signal intensity *x_ij_* in time sample *i* of voxel *j* is thus a weighted sum (linear mixing) of the unobserved source signals in the voxel, i.e. . With iterative optimising of an* unmixing matrix***W** = **A**^-1^, we obtain **S** = **WX** with mutually independent rows using e.g. the* InfoMax* algorithm [84,86]or the* FastICA* algorithm [87]. No noise is included in the model. Typically, the spatial maps are transformed to have zero mean and unit variance (Z-scores), thresholded at some level (e.g. |Z| > 2.0), and mapped back to 3-D image space, color-coded, and superimposed on a coregistered anatomical image for visual assessment of the ICA component.

It can be shown that the independent components are identifiable up to a permutation and scaling of the sources under the assumption that at most one of the sources s*_k_* = [*s_k_*_1_,..., *s_kn_*] (*k* = 1,... ,*p*) is Gaussian, and that the mixing matrix **A** is full rank. The assumption that the mixing matrix is square i.e. the complete case with as many mixtures (*M*) as sources (*K*), can be relaxed [88-90]. Important challenges are to reduce large fMRI data sets for ICA [91] and to estimate the number of independent components in the data [92]. Calhoun and coworkers [93] has also introduced an approach (GIFT) for drawing* group inferences* using ICA of fMRI data from many subjects, and Beckmann et al. [94] have constructed a "tensor probabilistic ICA" for multisubject fMRI analysis. These extensions have widened the scope and popularity of ICA for fMRI studies, especially group studies of resting state networks and the DMN (see [95] for a comparative framework with these approaches). Furthermore, ICA methods has also been applied to EEG recordings and combined fMRI-EEG data [96], with a large potential for integrated analysis of electrophysiological recordings (with high temporal, low spatial resolution) and BOLD image acquisitions (with high spatial, low temporal resolution). Recently, it has been argued that independence is not the right mathematical framework for blind source separation in fMRI and that representations in which the fMRI signal is spatially sparse are more promising [97], and in their simulation studies Daubechies et al. demonstrated that the ICA algorithms* InfoMax* and* FastICA* select for such sparsity of components rather than spatial independence.

### Theory of dynamical systems and neurodynamics

*... Neurodynamics has a peculiar property described as the "edge of stability" or "metastability". Accordingly, the brain as a complex dynamic system is in perpetual movement from one state to another. When the brain reaches a dominant state, it does not rest there, rather it immediately moves on and decays into an unordered state, only to emerge a moment later to another prominent state. Freeman has identified neurophysiological correlates of this metastable wandering along the landscape of brain dynamics in the form of spatio-temporal patterns of oscillations, sudden jumps or phase transitions of local field potentials. (From *[38]*)*

**Figure 3:**
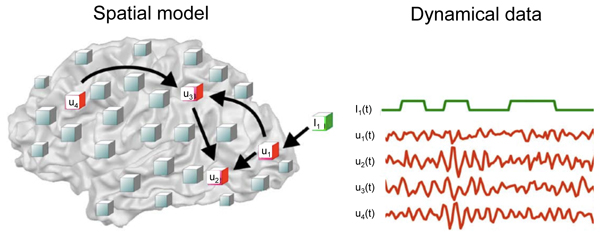
**Functional MRI voxel time courses and spatio-temporal activity patterns** in the context of experimental neurodynamics (modified from [155]).

To bring functional MRI into the domain of experimental neurodynamics (cf. Fig. 3), we can: (i) regard voxel time courses as the given spatio-temporal observations, or (ii) incorporate a "higher level" spatial representation based on anatomically meaningful regions (cf. the 38 cortical and 2 subcortical regions defined in [43]), or (iii) incorporate regions that are derived from spatial ICA analysis, or from cross-correlation clustering and graph theory (pionered by [98]), or from structural connectivity maps obtained with diffusion tensor MRI recordings and fiber tracking.

Due to the coarse spatial resolution of fMRI (for whole brain human studies voxel size is typically limited to ≥ 1µl) and low temporal resolution (typically ≥ 1000 ms), these measurement techniques will only be able to consider the granularity of 'mesoscopic brain dynamics', i.e. (nonlinear) dynamics and neural activity at spatial scales between a few millimeters and the entire brain, and temporal scales of a few hundred milliseconds to seconds (cf. [99]). Moreover, mesoscopic brain dynamics is characterized by “*its high complexity, often involving oscillations of different frequencies and amplitudes, perhaps interrupted by chaotic or pseudo-chaotic irregular behaviour. The mesoscopic brain dynamics is affected by the activity at other scales. For example, it is often mixed with noise, generated at a microscopic level by spontaneous activity of neurons and ion channels. It is also affected by macroscopic activity, such as slow rhythms generated by cortico-thalamic circuits or neuromodulatory influx from different brain regions*” [100]. As indicated above, there are a number of naturally occurring 'noise sources' in the brain (related to e.g. channel gating, ion concentrations, membrane conductance, and synaptic transmission [33]) that will influence signal measurements at mesocopic and macroscopic scales ( [101]). During the last years, the importance of noise in neural systems has been put in the context of 'stochastic resonance' – and before we deal with a classical framework for neural dynamics (the Wilson-Cowan model), we will present this highly interesting and relevant phenomenon.

#### Stochastic resonance

**Stochastic resonance** (SR) is* "a mechanism by which a system embedded in a noisy environment acquires an enhanced sensitivity towards small external time-dependent forcings, when the noise intensity reaches some finite level" *[102]. This SR effect introduces a new role of noise processes, not only as a detrimental nuisance, but as a constructive component in a broad range of natural and man-made systems. The mechanism was first observed and reported in climate research by Benzi et al. [103] in the early 1980's to explain the almost periodic recurrences of ice ages on the earth.

Mathematically, the SR phenomenon can be captured in a bistable nonlinear dynamical system where a one-variable formulation could be [104]:(1)

where the* state variablex*(*t*), interpreted as e.g. the position of a Brownian particle in a bi-stable potential *U* : ℝ → ℝ, is subject to both a weak periodic forcing parameterized with amplitude *A*, frequency *ω* and initial phase *ϕ*, and a noise process *ξ*(*t*). In our example *U*(*x*) can be the reflection-symmetric quartic potential  , with minima *x_m_* located at ±1 and with height of the potential barrier between the two stable minima equal to  . The noise term *ξ*(*t*) in the* stochastic differential equation* (1) is typically a zero-mean Gaussian where the SR mechanism is related to its autocorrelation function 〈*ξ*(*t*), *ξ*(0)〉, which could be on the form  where *τ* is* correlation time* and *D* is* intensity* of the colored noise. The effect of SR can be enhanced by introducing an ensemble of stochastic resonators. In this case, a weak periodic signal can induce large-scale stochastic synchronization and self-organization (Ch. 3 in [34]).

More recently, SR has been introduced to explain behavior in biological systems (see [105], 106 for reviews), including single neurons and large-scale brain systems (e.g. [107]). Neural synchrony, noise, and SR has also been applied to the modeling of attention and consciousness [108], and how age-related neuromodulatory deficiencies may contribute to increased neuronal noise leading to less robust information processing in aging neurocognitive systems [109,110]. Most interestingly, the stochastic resonance mechanisms has recently been proposed to explain resting state results in connectivity data from BOLD fMRI experiments [43].

**Figure 4:**
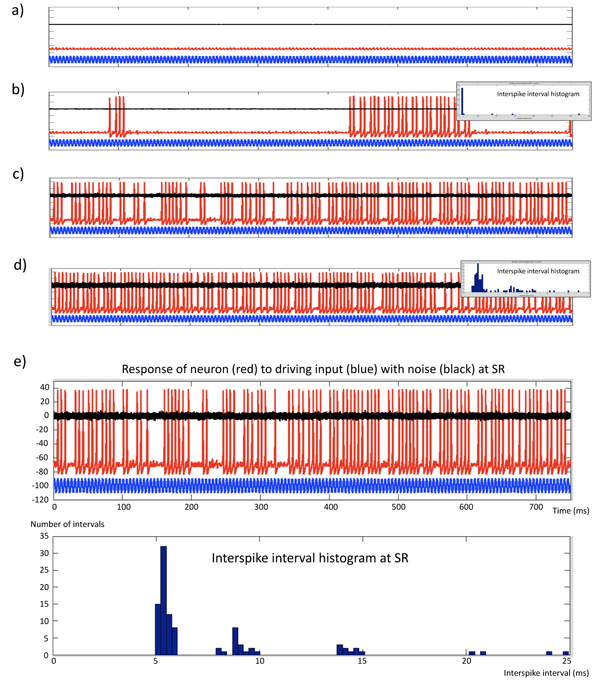
**Stochastic resonance** in a simple neuronal system with an oscillating driving force and a noise term. The simulated neuron is modeled with Hodgkin-Huxley dynamics [112] and is stimulated by a sinusoidal current I at a frequency 200 Hz and amplitude 0.035 (a.u.) with added zero mean Gaussian noise with standard deviation SD. At low noise levels (SD less than 0.01), spike threshold is not attained. With increased noise (SD = 0.04) irregular spike bursts occur. At a narrow noise band, stochastic resonance occur with inter-spike interval (ISI) at about 5 ms and integral multiples of that period. We see that the neuron can miss four or more periods between spiking, but keep firing at integral multiples of the 5 ms stimulus period. In the simulations, one could also show that (low and moderate) noise alone will not be sufficient to drive the spiking in the absence of the periodic stimulus. In this simulation we observe that sub-threshold noise can increase the sensitivity of the neuron to periodic stimulation - that be sensory input or input from a neuronal rhythm generator. This illustrates stochastic resonance. a) Insufficient noise level (SD = 0.01) to trigger spikes. b) Low noise level (SD = 0.04) - infra SR. c) Stochastic resonance, SR (SD = 0.19). d) High noise level (SD = 0.30) - supra SR. e) Magnification of the SR condition in c). Blue trace = oscillating driving force; Black trace = input noise; Red trace = response (firing pattern) of the simulated neuron at the given noise level. The inserts in b) and d) show the corresponding inter-spike interval histograms.

As an illustrative example of stochastic resonance in basic neurophysiology, we will briefly present a simulation study of the spiking behavior in a neuron with Hodgkin-Huxley (HH) dynamics [111], where the firing of action potentials is driven by a sinusoidal external force and Gaussian noise. The simulation code we are using is modified from the exposition on nonlinear neurodynamics by H.R. Wilson [112] (HHnoise.m), using a fourth order Runge-Kutta solver with constant step size to solve the Rinzel approximation to the HH equations:

For the simulations we used time increment Δ*t* = 0.02, and *a* = 17.81, *b* = 47.71, *c* = 32.63, *d* = 26. The equilibrium potentials were *E*_Na_ = 0.55 and *E*_K_ = –0.92. The neural capacitance was *C* = 0.8, and the recovery time constant *τ_R_* = 1.9 ms. The stimulating oscillating current was given as *I*(*t*) = *A* sin(*ωt* + *ϕ*) with amplitude *A* = 0.035 and initial phase *ϕ* = 0, were the *ω* was fixed to give an angular input frequency of 200 Hz. We adjusted zero mean noise level *η* with standard deviations (SD) in steps of 0.01 in the range [0.0, 0.5] to observe the effect of noise level of the system and the occurrence of stochastic resonance. The results from this simulation study are given in Fig. 4, where Figs. 4c &4e depict SR with clustering of spike intervals corresponding to integral multiples of the principal inter-spike interval of about 5 ms.

#### Linking fMRI and neurodynamical modeling

The theory of dynamical systems has been applied to fMRI in different settings [113-118]. To explore a particularly interesting link between fMRI and neurodynamical modeling in more detail, we adopt the approach taken by Deco et al. [43]. Here, the dynamics of each 'node' (to be interpreted below) is captured by a mean-field type rate model, expressing the coupling between excitatory and inhibitory nodes in the network. This is very similar to the classical* attractor neural network* (ANN) model stated by Amit [37] and others.

More specifically a Wilson-Cowan [119] type neural network dynamics is introduced, following [120,121](2)

where *u_i_* denotes the activity of the *i*-th 'neuron'* (neural mass,* or enumerated spatial localization *i* = 1,..., *n* in the brain); *α* is inverse relaxation time; *W_ij_* is the 'synaptic' or* connection strength* between locations *i* and *j* (i.e. the connectivity matrix); *I_i_* is the external input to location *i*; and *ξ_i_* is random noise related to *i*. The nonlinear (e.g. sigmoidal) function Θ(*x*) limits the growth of *x*, accounting for saturation of 'firing rates' or local neural mass activity. In the* 'resting state',* external inputs to the network nodes vanish, i.e. *I_i_* ≡ 0, where we also assume the noise terms *ξ_i_* are Gaussian *N*(0, *σ_i_*), and that saturation can be ignored, i.e. Θ(*x*) ≡ *x*. With these assumptions, the coupled stochastic ODE's (2) can be discretized in time, with time step Δ*t*, to obtain(3)

which can be written on matrix form

***u***(*t* + Δ*t*) = **A*u***(*t*)+***ξ***(*t*) (4)

where **A** = (1 – αΔ*t*)1 + **W**Δ*t*;***ξ*** = *N*(0,***σ***)(*t*)Δ*t*, and *N*(0,***σ***)(*t*) denotes the noise column vector at time point *t*. The temporal averaged covariance matrix of the resting state time courses is *C* = 〈u(*t*) • u(*t*)*^T^*〉, and the eigenvectors v*_k_* of the real-valued symmetric matrix *C* are the principal components or the dominant patterns or modes of the resting state activity, i.e.  . In [120] it is shown that the covariance matrix of the resting state activity is determined by the covariance matrix of the intrinsic noise and the eigenvalues of the connectivity matrix **W**. As the overall weight of each mode in the resting state activity is given by the magnitude of the associated eigenvalue λ*_k_*, there is link between the dominant patterns and the concept of* attractor* in neural dynamics [120]. Morover, Galán [120] provides arguments that the resting state neural activity 'fluctuates most of the time around the* basin of attraction* ( [37,122]) of the dominant pattern', and that 'the amount of time spent around the basin of attraction of the remaining principal components is proportional to the magnitude of their eigenvalues'.

In their simulation study of resting brain fluctuations, Deco and co-workers [43], using the above Wilson-Cowan model with 38 cortical and 2 subcortical thalamic nodes and a realistic neuroanatomical connectivity matrix and signal transmission delay between nodes in the network, were able to reproduce typically occurring resting state fMRI brain dynamics. In their synchronization analysis (employing the Kuramoto synchronization index [123]) of simulated neuroelectric activity in each of two individual community clusters (the occipital-temporal-prefrontal community, and the sensorimotor-premotor community), using internode communication velocity of *v* ~ 1.65 m/s and global coupling strength of *α* ~ 0.007, a global slow 0.1 Hz) oscillation at rest could emerge from a network built up with simple fast oscillators in the γ-band of 40 Hz. This underscores the role of neural synchronization as a mechanism for the emergence of ultraslow fluctuations in e.g. the* default mode network* as observed in resting state BOLD fMRI. Moreover, Deco et al. also observed a* stochastic resonance* effect for the same level of fluctuations that revealed optimal emergence of 0.1 Hz global slow oscillations, with the occurrence of anti-correlated spatiotemporal patterns (as reported in fMRI) in both communities – without the use of (unrealistic) long-range inhibition. From their work, they conclude that* "the particular dynamics of the intrinsic properties of the brain are useful for keeping the system in a high competition state between the different subnetworks that later are used during different tasks. ... In this way, a relatively small external stimulation is able to stabilize one or the other subnetwork giving rise to the respective evoked activity. So, the anticorrelated fluctuating structure of the subnetwork patterns characteristic of the resting state is particularly convenient for that."*

#### Linking consiousness and neurodynamics

The field of neurodynamics has also had impact on the theory of consciousness [38,41,124-127] (so has stochastic resonance [32,108,128]). A prominent example is the* 'dynamic core hypothesis'* presented in a seminal paper by Tononi and Edelman [129], and later refined and extended [127,130,131]. Based on modeling concepts from statistical physics and information theory, together with accumulated empirical findings in the history of clinical and experimental neuroscience, they propose the following:

• A group of neurons can contribute directly to conscious experience only if it is part of a distributed functional cluster that achieves high integration in hundreds of milliseconds.

• To sustain conscious experience, it is essential that this functional cluster be highly differentiated, as indicated by high values of complexity.

• A large cluster of neuronal groups exist that together constitute, on a time scale of hundreds of milliseconds, a unified neural process of high complexity – termed the **dynamic core**, where

– its participating neuronal groups are much more strongly interactive among themselves than with the rest of the brain

– its global activity patterns must be selected within less than a second out of a very large repertoire

– deliberately does not refer to a unique, invariant set of brain areas (e.g. prefrontal, extrastriate, or striate cortex) and,

– can transcend traditional anatomical boundaries and may change in composition over time

An other interesting neurodynamics theory of consciousness is the* COrollary Discharge of Attention Movement* (CODAM) model of J. G. Taylor and co-workers [132,133], based on application of engineering control theory and artificial neural networks. The CODAM framework is claimed to have considerable experimental support for certain of its modules. Support that comes from both brain imaging, ERP studies, and single cell experiments, as well as transcranial magnetic stimulation (TMS) that enables non-invasive, temporarily 'knock out' of brain circuitry in operator-defined cortical regions. In this model, the so-called 'working memory corollary discharge (WMcd) buffer' is the most important element in the circuitry to create consciousness. The framework consists of an input module (for pre-processing in low-level visual cortex), the object map (where object codes are stored), the inverse model controller (IMC) which is a generator of the signal to move the focus of attention in lower cortices, the corollary discharge module where a copy of the attention movement signal is stored temporarily, the working memory (WM) holding an estimate of the attended target representation, and the monitor producing an error signal given by the difference of the required goal signal and that produced by the corollary discharge module as a predictor of the attended next state or of the working memory module activation (cf. [133]). So far, very little work has been done to put fMRI experiments into the CODAM framework, which might have been due to difficulties of interpreting fMRI data in terms of timing and inhibition ( [134]).

As resting state fMRI networks revealed by e.g. independent component analysis might represent* neural mass activity* related to the state of consciousness ( [17]), further empirical investigations and numerical simulations of distinct network components and their time courses, IC_1_(*t*), ..., IC*_n_*(*t*), should be performed in the framework of Wilson-Cowan type neural network dynamics ( [43,121]). Moreover, measuring fMRI *resting state functional connectivity* (rsFC) and* structural connectivity* (SC), using DTI or diffusion spectrum imaging tractography at high resolution in the same individuals, it has been shown that the organizations of SC and of rsFC are strongly interrelated [121,135] 135. In [121] the authors applied Eq. (4) to model macroscopic cortical dynamics within segmented cortical parcellations (*n*=998), where the inter-nodal interaction efficacy matrix **W** in the generalized coupling matrix **A** = (1 – *α*Δ*t*)1 + **W**Δ*t* was the resampled fiber strengths obtained from diffusion spectrum imaging tractography. Such approaches might open up a new opportunity to combine mutlimodal MRI and neurodynamics in the study of consciousness.

In the following experiment, we illustrate the first steps of deriving time course information from resting state fMRI data that can be further analyzed in such modeling frameworks.

### An experimental resting state fMRI illustration

As an illustrative example, we report data from one subject (651) in a cohort of 100 healthy elderly people that were scanned with a 1.5 Tesla GE Signa Excite scanner equipped with an eight-channel head coil as part of a larger longitudinal study of* cognitive aging *[136-139], where our laboratory was responsible for the image analysis.

#### Data acquisition

While the subject was lying comfortably in the scanner with eyes closed, head fixed with noise-protecting ear-pads, and asked not to fall asleep, we used an EPI gradient echo pulse sequence (TR / TE / FA = 2 s / 50 ms / 90*^0^*); 64 × 64 acquisition matrix, 25 slices, 256 time frames, with voxelsize = 3.75 × 3.75 × 5.5 mm^3^ and time resolution of 2 s to obtain "resting state" data. In addition, the imaging protocol consisted of an alternating left and right hand finger-tapping fMRI paradigm, two anatomical 3D T1-weighted scans for brain segmentation with FreeSurfer (http://surfer.nmr.mgh.harvard.edu), and DTI acquisitions (TR / TE / FA = 7900 / 104.8 / 90; 25 b=1000, 5 b=0; 25 slices; voxelsize = 1.88 × 1.88 × 4.0 mm^3^) for assessment of white matter integrity and fibertracking with TrackVis (http://trackvis.org).

#### Data processing

**Figure 5:**
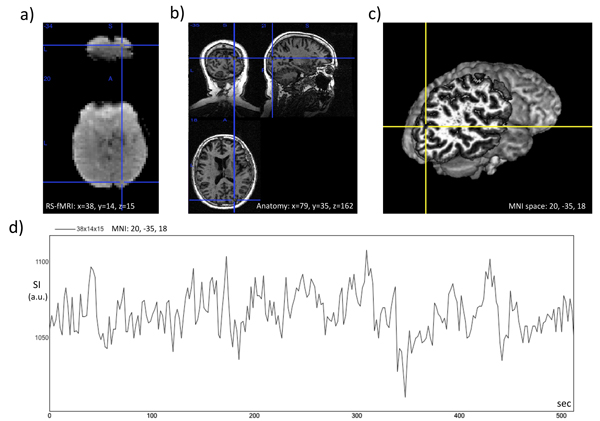
**Voxel time course in right cerebral hemisphere from subject 651.** a) Time frame from the resting state fMRI recording, where a voxel-of-interest in the visual area of the occipital lobe is marked with a cross-hair. b) Corresponding voxel in the 3D Tl-weighted anatomical image. c) Same voxel in the volume-rendered 3D anatomical image obtained with MRIcron (http://www.sph.sc.edu/comd/rorden/MRicron) d) Plot of signal intensity (S.I.) versus time for the same voxel.

**Figure 6:**
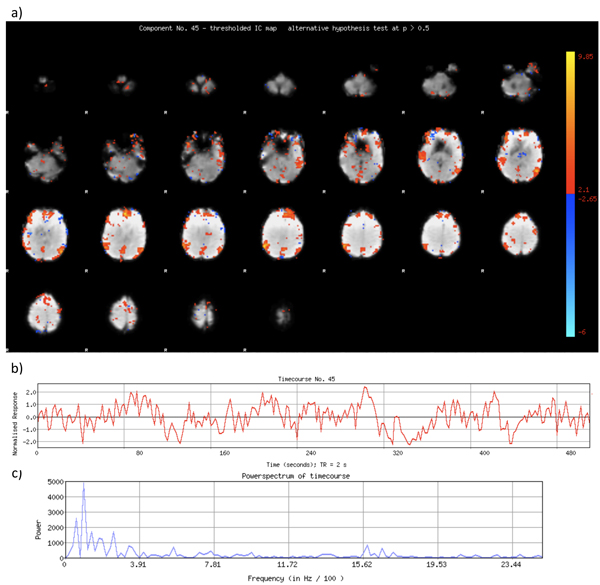
a) Spatial extension of MELODIC component no. 45, a thresholded IC map, representing the* default mode network.* b) The temporal mode or timecourse of component no. 45. c) Powerspectrum of timecourse belonging to component no. 45. Note the peaks in the low frequency band ~ 0.01 – 0.02 Hz.

Image processing and analysis of the "resting state fMRI" data was carried out using Probabilistic Independent Component Analysis [85] as implemented in MELODIC (Multivariate Exploratory Linear Decomposition into Independent Components) Version 3.09, part of FSL (FMRIB's Software Library, http://www.fmrib.ox.ac.uk/fsl) [29]. The following data pre-processing was applied to the input data: masking of non-brain voxels; voxel-wise de-meaning of the data; normalization of the voxel-wise variance; Pre-processed data were whitened and projected into a 60-dimensional subspace using probabilistic Principal Component Analysis where the number of dimensions was estimated using the Laplace approximation to the Bayesian evidence of the model order [85,140]. The whitened observations were decomposed into sets of vectors which describe signal variation across the temporal domain (time-courses) and across the spatial domain (maps) by optimizing for non-Gaussian spatial source distributions using a fixed-point iteration technique [87]. Estimated Component maps were divided by the standard deviation of the residual noise and thresholded by fitting a mixture model to the histogram of intensity values [85]. Figure 5 shows recorded images and a voxel time course from the subject, while Fig. 6 depicts MELODIC component no. 45, representing the *default mode network*.

**Figure 7:**
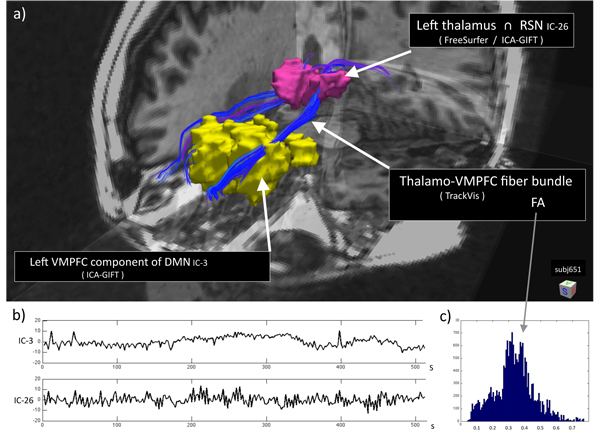
**Integration of structural connectivity (DTI) and functional resting state networks (fMRI).** All derived information from the multimodal MRI examination (subj651) was obtained automatically with different freely-available analysis tools. Coregistration was performed in Matlab. a) Tl-weighted anatomical 3-D image superimposed with: (i) anatomical region* (thalamus)* segmented by FreeSurfer; (ii) resting state networks (RSNs) derived from ICA analysis (GIFT) of the BOLD fMRI recordings; (iii) white matter fiber tracts (TrackVis) between these regions of interest (ROIs). Yellow blob is the* ventromedial prefrontal cortex* (VMPFC) component of the default mode network (DMN). b) Corresponding time courses (256 frames; TR= 2 s) of the resting state components. Upper trace is IC-3 (DMN). Lower trace is IC-26 located in the central thalamic region (not part of the DMN). c) Histogram of the FA values, reflecting white matter integrity and structural connectivity along the fiber tracts between the two regions: (i) spatial intersection between anatomical thalamus and the RSN defined by IC-26, and (ii) the VMPFC component of DMN defined by IC-3. [Courtesy of Martin Ystad, Tome Eichele, Erlend Hodneland, and Judit Haasz]

A more advanced processing scheme of the multimodal MRI acquisitions from the same subject (651), combining both structural connectivity information from DTI recordings and functional connectivity from resting state fMRI recordings, is shown in Fig. 7. [The original acquisitions Anatomyi.nii, Anatomy2.nii, DWI.nii, bvec.dat, bval.dat, Resting.nii, Fingertapping.nii are available in NIFTI-1 format from the author; see also http://sites.google.com/site/hufy372 ]

## Concluding remarks and perspectives

During the last few years, functional MRI has been introduced as a new* tool to measure consciousness,* both in a clinical setting and in basic neurocognitive research [1,2,7,8,18,141-153], and advanced methods and theories (e.g. computational neuroimaging) has arrived the field of fMRI [114,121,154-158]. The aim of this paper has been to present the great excitement (and limitations) regarding the use of fMRI as a tool for the study of one of the Big Questions - the neural correlates of consciousness.

To end up with a more balanced picture regarding the measurement of NCC, we have also to address *electrical neuroimaging* (e.g. [159]) and the assessment of neurodynamics at* subsecond time scale* - not yet possible with MRI. Up to now, electrophysiology has had much greater impact than fMRI on the study of consciousness, both in the clinics [160-162] and in basic research [163,164]. However, during the last years the technology for obtaining simultaneous recordings of multichannel electroencephalography (EEG) and BOLD fMRI acquisitions has been developed. This technology, involving both hardware solutions and clever data analysis, exploits and joins the advantages of each technique - the high spatial resolution of fMRI and the high temporal resolution of EEG. Due to these developments there are an increasing number of investigations being performed, both in the clinics (e.g. epilepsy) and in neurocognitive research that uses simultaneous EEG-fMRI recordings to disentangle brain processes and spatio-temporal activity patterns [75,96,165-177]. Recently, Musso et al. [178], using a novel EEG-fMRI analysis approach to explore resting-state networks, were able to show that the information contained within EEG microstates on a millisecond timescale was able to elicit BOLD activation patterns consistent with well known RSNs - "opening new avenues for multimodal imaging data processing". Finally, direct mapping of neuronal activity using MRI detection has been demonstrated [179-182], but seems not to be very promising, or of practical value, so far [183].

To conclude this paper, we will briefly bring up some themes and challenging topics that need to be clarified, or incorporated into experimental procedures and modeling frameworks:

• The emergence of human consciousness [184]

• Consciousness without a cerebral cortex? [185] Is your brain really necessary? [186]

• The possibility or plausibility of* quantum information processing* capabilities of neuronal microtubules (MT) and relation to consciousness, with proponents [187-193] and opponents [195-197]

• Different time scales in the brain [198,199]

• Fast dynamics in local and large-scale networks [200], and hierarchy of attractors [201]

• Direct correlations between the BOLD response and neuronal electrophysiology [202,203]

• Resting state fMRI - "spatially sparse" rather than "spatially independent" componets? [97]

• Mind reading using fMRI time courses [14,183,204-207]

• Consciosness and unilateral neglect [208] – the role of fMRI ? [209,210]

• Graded consciousness and fMRI [1,7,8,67,70,142,144,211-219] and unconscious cognitive control [146,220].

Finally, within frameworks of* emergentism, global workspace theory, dynamic core hypothesis,* and the *CODAM model* we believe the combination of (i) resting state fMRI* (functional connectivity),* (ii) diffusion tensor MR imaging* (structural connectivity),* and (iii)* neurodynamical modeling* will bring a new set of tools to the study of consciousness and cognition – that be during early development, during aging and neurodegeneration, and in altered brain states such as anesthesia, sleep, and meditation.

## List of abbreviations used

4-D: four dimensional (3-D + time); ANN: attractor neural network; BOLD: blood-oxygenation-level-dependent; BSS: blind source separation; CODAM: COrollary Discharge of Attention Movement; DMN: default mode network; DTI: diffusion tensor imaging; EEG: electroencephalography; ERP: event related potentials; FA: flip angle (in MRI); fMRI: functional magnetic resonance imaging; GW: global workspace (theory); HH: Hodgkin-Huxley (dynamics); IC: independent (spatial) component; ICA: independent component analysis; MT: microtubule; NCC: neural correlates of consciousness; NMM: neural mass model; ODE: ordinary differential equation; PET: positron emission tomography; rsFC: resting state functional connectivity; RSN: resting state network; SC: structural connectivity; SDE: stochastic differential equation; SR: stochastic resonance; TE: echo time (in MRI); TMS: transcranial magnetic stimulation; TR: repetition time (in MRI); VMPFC: ventromedial prefrontal cortex; WM: working memory

## Competing interests

The author declare that he has no competing interests.

## Authors information

Arvid Lundervold has a BSc in mathematics and philosophy from the University of Oslo (1975) and got his medical degree (MD) from the same university (1982). While in Oslo he also worked with experimental epilepsy (the hippocampal slice preparation) at the Institute of Neurophysiology and at the National Hospital. He obtained his PhD ("Multispectral Analysis, Classification and Quantification in Medical Magnetic Resonance Imaging") at the University of Bergen in 1995. He has professional experience in medical informatics from the National Hospital in Oslo (1984-1988), and as research scientist at the Norwegian Computing Center, Image Analysis and Pattern Recognition group (1989-1994), before he came to the University of Bergen in 1994. He his currently a Professor in medical information technology at the University of Bergen, Department of Biomedicine, and head of the Neuroinformatics and Image Analysis Laboratory. Lundervold is also affiliated with the Department of Radiology, Haukeland University Hospital. He has published more than 100 papers and conference reports related to medical image analysis, pattern recognition, and neuroinformatics. Lundervold has supervised or co-supervised more than twenty Master and PhD students with their basic training from mathematics, computer science, medicine, or physiology. He has participated in the European COST B11 action ("Quantitation of Magnetic Resonance Image Texture"), the COST B21 ( "Physiological Modelling of MR Image Formation"), and the COST BM0601 ("NEUROMATH - Advanced Methods for the Estimation of Human Brain Activity and Connectivity"). He is on the editorial board of* Computerized Medical Imaging and Graphics* and of* Frontiers in Neuroinformatics.* Lundervold is member of the Norwegian Medical Association, the International Society for Magnetic Resonance in Medicine, the IEEE Computer Society, and the American Mathematical Society.
